# Coumarin Derivative Hybrids: Novel Dual Inhibitors Targeting Acetylcholinesterase and Monoamine Oxidases for Alzheimer’s Therapy

**DOI:** 10.3390/ijms252312803

**Published:** 2024-11-28

**Authors:** Teresa Żołek, Rosa Purgatorio, Łukasz Kłopotowski, Marco Catto, Kinga Ostrowska

**Affiliations:** 1 Department of Organic and Physical Chemistry, Faculty of Pharmacy, Medical University of Warsaw, Banacha 1, 02-097 Warsaw, Poland; lukasz.klopotowski96@gmail.com (Ł.K.); kostrowska@wum.edu.pl (K.O.); 2 Department of Pharmacy-Pharmaceutical Sciences, University of Bari Aldo Moro, Via E. Orabona 4, 70125 Bari, Italy; rosa.purgatorio@uniba.it (R.P.); marco.catto@uniba.it (M.C.)

**Keywords:** coumarin derivatives, hAChE/hMAO-B dual-targeted inhibitor, Alzheimer’s disease, molecular docking, molecular dynamic simulation

## Abstract

Multi-target-directed ligands (MTDLs) represent a promising frontier in tackling the complexity of multifactorial pathologies like Alzheimer’s disease (AD). The synergistic inhibition of MAO-B, MAO-A, and AChE is believed to enhance treatment efficacy. A novel coumarin-based molecule substituted with *O*-phenylpiperazine via three- and four-carbon linkers at the 5- and 7-positions, has been identified as an effective MTDL against AD. Employing a medicinal chemistry approach, combined with molecular docking, molecular dynamic simulation, and ΔG_bind_ estimation, two series of derivatives emerged as potent MTDLs: 8-acetyl-7-hydroxy-4-methylcoumarin (IC_50_: 1.52–4.95 μM for hAChE, 6.97–7.65 μM for hMAO-A) and 4,7-dimethyl-5-hydroxycoumarin (IC_50_: 1.88–4.76 μM for hMAO-B). They displayed binding free energy (ΔG_bind_) of −76.32 kcal/mol (**11**) and −70.12 kcal/mol (**12**) against AChE and −66.27 kcal/mol (**11**) and −62.89 kcal/mol (**12**) against MAO-A. It is noteworthy that compounds **11** and **12** demonstrated efficient binding to both AChE and MAO-A, while compounds **3** and **10** significantly reduced MAO-B and AChE aggregation in vitro. These findings provide structural templates for the development of dual MAO and AChE inhibitors for the treatment of neurodegenerative diseases.

## 1. Introduction

Alzheimer’s disease (AD) is a deadly neurodegenerative disorder characterized by the progressive degeneration of neurons in the central nervous system [1]. The symptoms begin with memory loss, followed by progressive and irreversible cognitive impairments, a decline in language skills, severe behavioral abnormalities, and, ultimately, death [2]. Although AD was identified over a century ago, it remains incurable due to the incomplete understanding of its molecular etiology. To explain the mechanism of AD pathogenesis and occurring symptoms, two hypotheses including non-cholinergic and cholinergic interventions were created. The non-cholinergic hypothesis is characterized by intracellular deposits of τ-proteins, which affect intracellular transport and lead to cell death, and extracellular deposits of β-amyloid (Aβ) peptides, which are accompanied by oxidative stress and the inflammation of neurons. The cholinergic hypothesis emphasizes the deficit of neurotransmitter acetylcholine (ACh) in the brain regions of AD patients that leads to memory and cognitive impairments and supports that a decrease in the ACh hydrolysis by inhibiting acetylcholinesterase (AChE) can alleviate these symptoms [3,4,5,6,7]. It has been indicated that AChE contains two distinct binding sites: a peripheral anionic site (PAS) and a catalytic active site (CAS). AChE inhibitors that bind to either the PAS or both the CAS and PAS may simultaneously alleviate cognitive deficits and delay the neurodegenerative process by preventing the aggregation of Aβ peptides [8]. The current clinical treatment of this disease primarily targets the cholinergic hypothesis [9]. Therapeutic options include one *N*-methyl-D-aspartate (NMDA) receptor antagonist (memantine) and three hAChE inhibitors (donepezil, galantamine, and rivastigmine). However, due to the multifactorial nature of AD, these drugs provide only modest symptom relief and cannot reverse brain damage or prevent neuronal degeneration [10,11,12]. Considering the complex etiology of AD and the likelihood that ACh depletion is not the sole factor contributing to the disease, a more comprehensive approach known as the multi-target-directed ligand (MTDL) strategy has been proposed. The MTDL strategy aims to design a single molecule capable of simultaneously modulating multiple targets involved in the neurodegenerative cascade of AD with the goal of effectively altering the disease’s progression or potentially curing it [12,13].

Many research studies have indicated that other neurotransmitter systems, especially the dopaminergic, serotoninergic, and monoaminergic systems, also play a pivotal role in the pathogenesis and development of AD [14,15]. Brain monoamine oxidases (MAO-A and MAO-B) have garnered significant attention in recent years. MAO-A primarily degrades serotonin, adrenaline, and noradrenaline and is closely associated with depression. In contrast, MAO-B activity is elevated in AD patients, resulting in increased dopamine metabolism and the excessive production of hydrogen peroxide, which ultimately contributes to neuronal dysfunction [16]. Additionally, activated MAO-B can disrupt the cholinergic system, damage cholinergic neurons, and promote the formation of amyloid plaques [17]. As a result, designing MTDLs that simultaneously inhibit hAChE and hMAO-B or hMAO-A has emerged as a promising therapeutic strategy for neurodegenerative disorders, including AD. A lot of hAChE and hMAO-B inhibitors with good therapeutic effects have been reported [18,19,20]. Among the compounds identified, ladostigil, which was designed using a combination of two pharmacophores taken from rivastigmine and rasagiline is currently in phase IIb clinical trials [21], which encourages us to further search for other new multi-target molecules with hAChE and hMAO-B inhibitory activity.

One of the latest trends is the search for new anti-AD drugs among coumarin derivatives with piperazine moiety. This research was inspired by the observation that Ensaculin (KA-672) inhibits hAChE activity in vitro (IC_50_ = 0.36 μM) and slows or prevents progressive neurodegeneration [22]. Ensaculin is a coumarin derivative substituted with a piperazine moiety, which has also demonstrated memory-enhancing effects in passive and conditioned avoidance paradigms in both normal and artificially amnesic rodents [23,24]. Therefore, the combined effects of Ensaculin on neurotransmitter systems and its potential neurotrophic and neuroprotective properties make it an intriguing and promising candidate in the search for a drug to treat AD. Moreover, research has shown that Ensaculin exhibits low target organ toxicity, which is a significant advantage for AD treatment [25]. Based on the structural features of Ensaculin, several researchers have attempted to incorporate various structural moieties into the coumarin scaffold to develop hAChE inhibitors [22,25,26]. Coumarins with substitutions at the C-3 and/or C-4 positions were found to exhibit anti-AChE activities comparable to the reference drug, Donepezil. Substituents such as phenyl, methyl, carboxylic acid, ethyl ester, or acyl chloride at the C-3 position resulted in potent MAO inhibitors [24]. It is noteworthy that coumarin is an environmentally friendly heterocycle that is easy to functionalize, offering a high degree of potential chemical diversity [27]. Its planar backbone can be efficiently accommodated in the MAO-B catalytic site [28] and can interact with the peripheral anionic site PAS of hAChE [29]. Additionally, it can occupy the substrate cavity of hMAO-B, thus exhibiting potent hMAO-B inhibitory activity [30]. In turn, the nitrogen atom in the phenylpiperazine group acts as the positive charge center, which is a feature commonly found in many potent hAChE inhibitors. X-ray crystallographic studies of the hAChE-donepezil and hAChE-galantamine complexes confirmed that the positive charge center interacts with the catalytic site of hAChE, while the phenyl ring attached to the piperazine ring serves as the choline-binding site [24].

**Figure 1:**
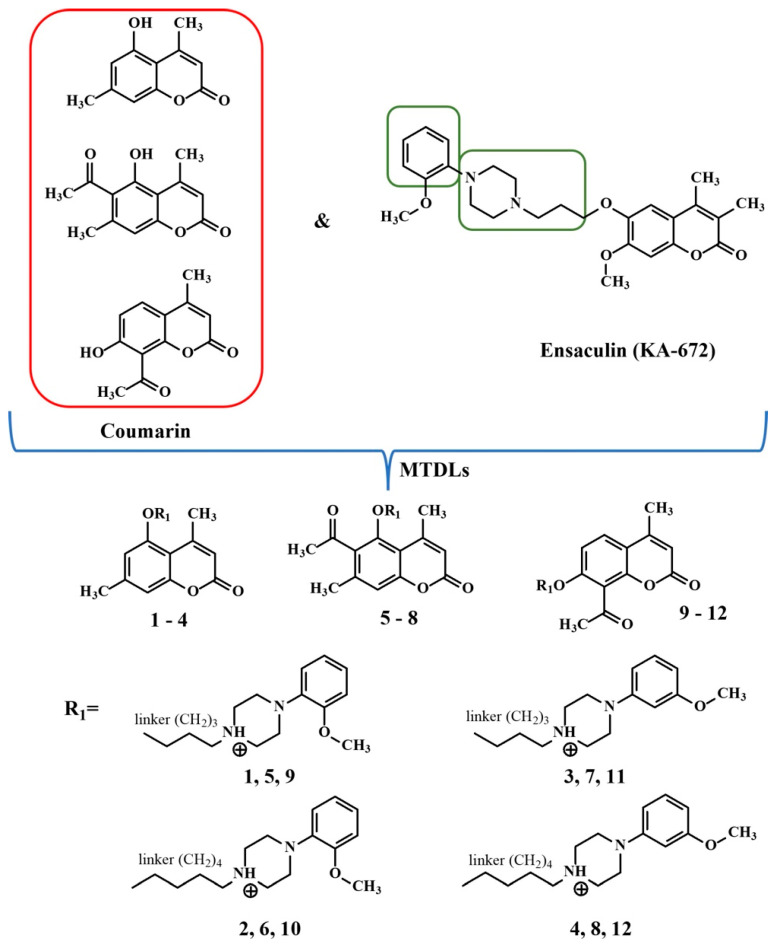
Design strategy for Ensaculin–coumarin hybrids.

Considering these factors, we developed small molecules with effective multi-target action against AD, validating their efficacy through in silico and in vitro studies. We investigated coumarin structures conjugated with 4,7-dimethyl-, 6- or 8-acetyl-, and phenylpiperazine via a flexible three- and four-carbon linker at the less explored positions C-5 and C-7 (Figure 1). The compounds were designed to incorporate coumarin structures known to exhibit MAO-B inhibition [28] and selected structural elements of Ensaculin for selective hAChE inhibition.

The designed series of coumarin–piperazine derivatives were synthesized and tested in vitro to evaluate their ability to inhibit hAChE and hMAO enzymes. To rationalize the molecular basis for the observed biological activity, molecular dynamics (MD) simulations in an explicit solvent system were performed to assess the dynamic behavior, stability, and conformational transitions of the new hybrid compounds within the binding sites of hAChE, hMAO-A, and hMAO-B.

## 2. Results and Discussion

### 2.1. Synthetic Coumarins

**Scheme 1:**
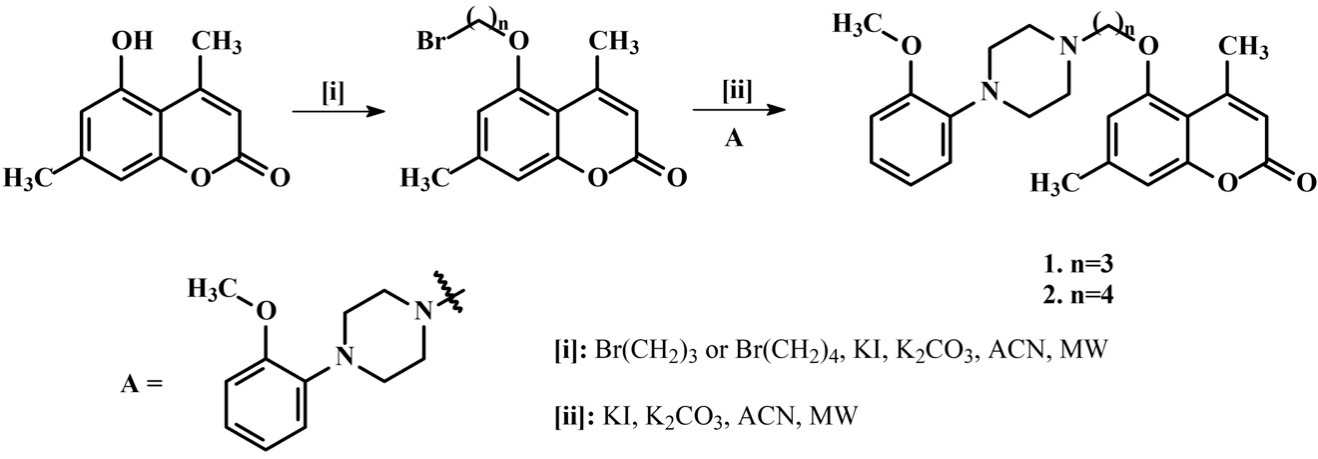
Synthetic route of new coumarin derivatives **1** and **2**.

The starting coumarins, 5-hydroxy-4,7-dimethylchromen-2-one, 6-acetyl-5-hydroxy-4,7-dimethylchromen-2-one, and 8-acetyl-7-hydroxy-4-methylchromen-2-one, were re-synthesized according to previously published methods (Scheme 1) [31,32].

The reaction of starting coumarins with 1,3-dibromopentane or 1,4-dibromobuthane in acetonitrile in the presence of potassium iodide and potassium carbonate yielded 5-(3-bromopropoxy)-4,7-dimethyl-2H-chromen-2-one, 5-(4-bromobuthoxy)-4,7-dimethyl-2H-chromen-2-one, 6-acetyl-5-(3-bromopropoxy)-4,7-dimethyl-2H-chromen-2-one, 6-acetyl-5-(4-bromobuthoxy)-4,7-dimethyl-2H-chromen-2-one, and 8-acetyl-7-(3-bromopropoxy)-4-methylchromen-2-one and 8-acetyl-7-(4-bromobuthoxy)-4-methylchromen-2-one, as described in our previous works [33,34,35,36]. In the next step, the final compounds were synthesized according to a previously published study [33]. The synthesis of compounds **1**–**12** was carried out by reacting the bromoalkyl derivatives with the appropriate arylpiperazine: 4-(2-methoxyphenyl)piperazine or 4-(3-methoxyphenyl)piperazine in acetonitrile in the presence of potassium iodide and potassium carbonate. The synthesis process was monitored by TLC using silica gel plates (eluent (CHCl_3_: MeOH, 10:0.25)). All compounds synthesized in this work were prepared using a microwave reactor and purified using column chromatography with silica gel. Characterization of the compounds was performed using ^1^H NMR, ^13^C NMR spectroscopy, and HRMS spectrometry. Scheme 1 shows the synthesis of the two new compounds, **1** and **2**. The synthesis schemes and detailed data for compounds **3**–**12** are included in our previously published works [33,34,35,36]. The NMR spectra for two new compounds, **1** and **2**, are presented in Figures S1 and S2 (see Supplementary Materials).

### 2.2. Molecular Docking of Coumarin Derivatives with hAChE

Docking studies were performed to gain further insight into the binding mode of the coumarin derivatives and to elucidate the impact of structural modifications on the inhibitory activities of the compounds against hAChE. Several ligand-bound crystallographic structures of hAChE are available. For the docking procedure, the structure of 4EY7 was obtained from the RCSB as a complex bound with the inhibitor donepezil. To validate the docking protocol, the crystal structure of donepezil was re-docked onto the target enzyme, and the RMSD value was 0.65 Å, demonstrating the predictive accuracy of the method used.

**Figure 2:**
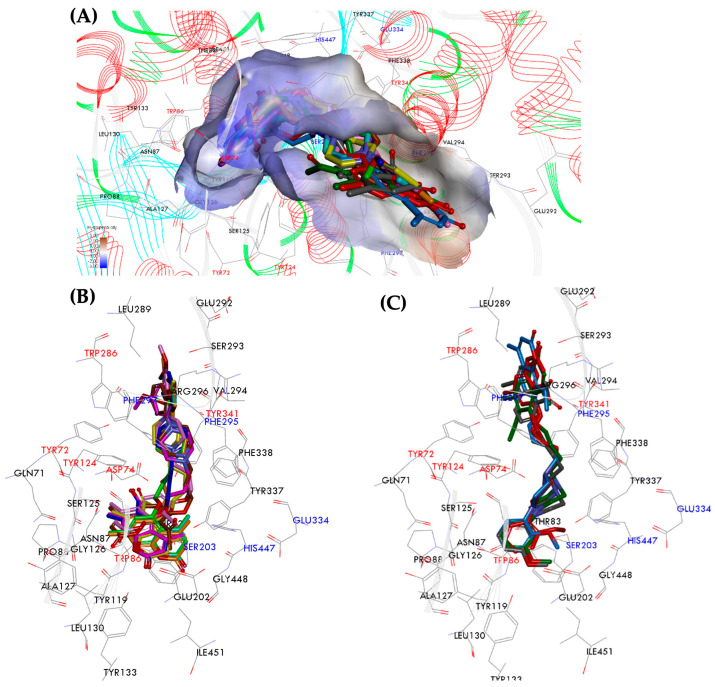
Superimposed coumarin derivatives docked into the binding pocket of hAChE. (**A**) The hydrophobic and hydrophilic amino acid residues surround the ligands. Surface hydrophobicity was depicted by the shaded colors: brown—the hydrophobic and blue—the lipophilic regions. (**B**) Shared binding orientation of compounds **1** (green), **3** (turquoise), **4** (orange), **5** (dark blue), **6** (yellow), **7** (dark pink), **9** (brown), and **11** (pink). (**C**) Superposition of compounds: **2** (red), **8** (dark green), **10** (blue), and **12** (gray).

As shown in Figure 2, all coumarin derivatives (**1**–**12**) were found to fit in the gorge of hAChE formed by CAS and PAS and surrounded by the residues Gln-71, Thr-83, Asn-87, Pro-88, Ser-125, Ala-127, Leu-130, Gly-148, Gln-291, Glu-292, Ser-293, Val-294, Tyr-337, and Ile-451, revealing a similar binding orientation as that of reference inhibitor donepezil [37]. In the hAChE, the CAS region is surrounded by Trp-86, Tyr-119, Tyr-124, Tyr-133, Glu-202, Ser-203, Trp-439, His-447, and Tyr-449, together with a series of glycine residues (Gly-120, Gly-121, Gly-122, Gly-126, and Gly-448), and PAS is in contact with Tyr-72, Asp-74, Thr-75, Trp-286, Leu-289, Tyr-341, and Val-365 residues. The mid-gorge site of hAChE is characterized by Asp-74, Leu-76, Phe-295, Arg-296, Phe-297, and Phe-338. Its opening is about 10.6 Å wide (average distance between Leu-76 and Phe-297), while the entire gorge pocket is approximately 20 Å deep.

The predicted locations of the studied coumarin derivatives in the hAChE pocket are similar (see Figure 2A), and their interactions are primarily hydrophobic. In almost all the test compounds (**1**, **3**, **4**, **5**, **6**, **7**, **9**, and **11**), the coumarin moiety was bound to the CAS of the enzyme, while the rest of the molecule (composed of alkyl chains, piperazine, and benzene rings) was directed toward the “anionic” site (PAS), accounting for the differences in binding modes (Figure 2B). However, a few exceptions were found. Compounds **2**, **8**, **10**, and **12** (with the butylene chain) were well-accommodated in the gorge of the hAChE active site such that the coumarin moiety leaned toward the PAS of the enzyme (Figure 2C). Tested compounds were held in the binding site by a combination of hydrogen bonds, hydrophobic interactions, and van der Waals interactions with their surroundings. In the case of the hAChE model, for the best-docked positions of the coumarin derivatives, strong hydrogen bonding interactions were observed, except for compounds **9** and **7**. Specifically, interactions were observed with the residues Gly-120, Tyr-124, and Tyr-133 for compounds **5**, **6**, and **11**; Tyr-72, Tyr-133, and Ser-293 for compound **8**; Tyr-124, Ser-293, and Arg-296 for compound **10**; Tyr-133 and Phe-295 for compound **12**; Glu-202 for compounds **1**, **3**, and **4**; and Ser-293 for compound **2**. In addition, all compounds exhibited carbon–hydrogen bonds due to the presence of several residues, such as Asp-74, Gly-121, Tyr-124, Gly-126, Glu-202, Ser-293, Val-294, Arg-296, Gly-448, Tyr-337, Tyr-341, and His-447, in the vicinity of the ligand. Moreover, all the compounds showed hydrophobic interactions, such as π–π stacked, π–alkyl, alkyl, π–sigma, and π–cation. The π–π interaction with residues Trp-86 and Trp-286 was observed for compounds **2**, **6**, **11**, **8**, **9**, and **12**; the interaction with residues Leu-289 and His-447 was observed for compounds **2** and **10**; the interaction with residues Phe-297, Tyr-72, Tyr-337, and Val-291 was observed for compound **2**; and the interaction with residues His-447 and Tyr-337 was observed for compound **3**. The π–cation interaction was observed with Asp-74 and Tyr-341 for compounds **1**, **3**, **4**, and **6**. Additionally, the π–sigma interaction with Val-294 was observed for compound **4**; and the interaction with Tyr-341 was observed for compound **7**. The formation of unfavorable bonds was noticed for compound **9** between the carbonyl oxygen of the coumarin moiety and residue Gly-120. Thus, in most of the potent compounds, hydrogen bonding and hydrophobic interactions were primarily responsible for mediating inhibitory action against *h*AChE. Therefore, in further research, we focused our attention only on the binding mode of the most active inhibitors: compounds **10**, **11**, and **12**. The poses that demonstrated the strongest interaction energies were selected as the starting ligand structures for extensive molecular dynamics simulations and binding free energy calculations.

### 2.3. Molecular Docking of Coumarin Derivatives with hMAOs

**Figure 3:**
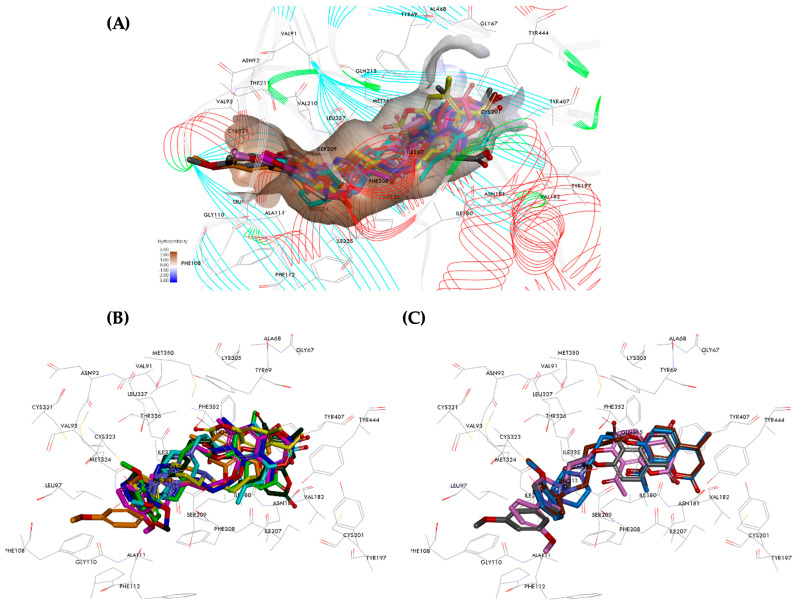
Superimposed coumarin derivatives docked into the binding pocket of hMAO-A. (**A**) The hydrophobic and hydrophilic amino acid residues surround the ligands. Surface hydrophobicity was depicted by the shaded colors: brown-hydrophobic region; blue-lipophilic region. The hMAO-A residues that make up the active site of the receptor are labeled in black. (**B**) Shared binding orientation of 5-hydroxy-substituted coumarins is displayed in **1** (green), **2** (red), **3** (turquoise), **4** (orange), **5** (dark blue), **6** (yellow), **7** (dark pink), and **8** (dark green). (**C**) Binding orientation of 7-hydroxy-substituted coumarins of **9** (brown), **10** (blue), **11** (pink), and **12** (gray) within the binding pocket.

**Figure 4:**
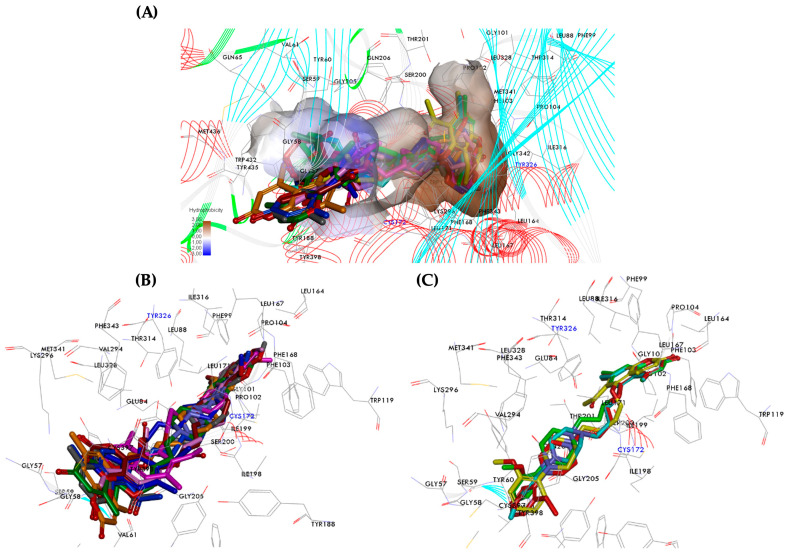
Superimposed coumarin derivatives docked into the binding pocket of hMAO-B. (**A**) The hydrophobic and hydrophilic amino acid residues surround the ligands. Surface hydrophobicity was depicted by the shaded colors: brown—hydrophobic region; blue—lipophilic region. (**B**) Shared binding orientation of compounds **4** (orange), **5** (dark blue), **7** (dark pink), **8** (dark green), **9** (brown), **10** (blue), **11** (pink), and **12** (gray). (**C**) Superposition of compounds: **1** (green), **2** (red), **3** (turquoise), and **6** (yellow).

Compounds **1**–**12**, most of which are 5-hydroxycoumarin derivatives, were also investigated for their inhibitory effects on monoamine oxidase. This study was conducted through the differential docking of the compounds into the structure of hMAO-A with the 7-methoxy-1-methyl-9H-β-carboline (harmine)-bound ligand (PDB code: 2Z5X) and hMAO-B in complex with 7-(3-chlorobenzyloxy)-4-(methylamino)methylcoumarin (PDB code: 2V61). The co-crystallized ligand, harmine, was re-docked into the binding site, where it binds hydrophobically with key amino acids of MAO-A, namely, Ile-180, Phe-208, Gln-215, Cys-323, Ile-325, Ile-335, Leu-337, Tyr-407, and Tyr-444. The re-docked harmine was superimposed onto its original position within an RMSD of 0.28 Å. The re-docked 7-(3-chlorobenzyloxy)-4-(methylamino)methylcoumarin was efficiently bound into the target macromolecule of 2V61, where it was superimposed onto the natively bound ligand with an RMSD of 1.68 Å. In both the binding pocket and the entrance cavity, residues Trp-119, Leu-164, Leu-167, Phe-168, Leu-171, Ile-198, Ile-199, Ile-316, and Tyr-326 were involved, while the substrate cavity was bordered by residues Tyr-60, Cys-172, Gln-206, Tyr-398, and Tyr-435. Based on the literature data, it was found that residues Tyr-326 (located in the side chain of MAO-B) and Ile-335 (located in the side chain of MAO-A), situated at the junction of the substrate and entrance cavity, play a crucial role in the substrate/inhibitor selectivity of the two MAO isoforms. Specifically, Tyr-326 prevents the binding of MAO-A selective inhibitors to the MAO-B active site, thus contributing to the selectivity toward either MAO-A or MAO-B [38,39]. The binding conformations of tested coumarins with MAO-A and MAO-B are shown in Figure 3 and Figure 4, respectively.

Evaluation of the virtual ligand–enzyme complexes of all coumarin derivatives within the catalytic site of MAO-A revealed that all tested compounds occupied the active site of MAO-A. They were accommodated within the binding pocket formed by the residues Tyr-69, Val-93, Leu-97, Phe-108, Arg-109, Gln-110, Ala-111, Ile-180, Asn-181, Tyr-197, Ile-207, Phe-208, Ser-209, Val-210, Thr-211, Gln-215, Cys-323, Ile-325, Ile-335, Thr-336, Leu-337, Met-350, Phe-352, Tyr-407, and Tyr-444 (Figure 3A). The best-ranked docking solutions of compounds **1**–**12** within the active site of MAO-A indicate that the coumarin moiety of all compounds binds in the polar region of the substrate cavity, near the FAD cofactor site. The rest of the molecule extends toward the entrance of the cavity space (Figure 3B,C). The residues Tyr-69, Phe-352, Tyr-407, and Tyr-444 formed the bottom of the aromatic cage, which determined the binding orientation of the compounds and consequently influenced their activity, binding affinity, selectivity, and stability. All compounds exhibited one or more hydrogen bonding interactions, except for compounds **1**, **2**, **3**, **4**, **5**, **6**, **7**, and **9**. Additionally, all the compounds demonstrated π–π interactions, except for the least active MAO-A inhibitors, i.e., compounds **2**, **5**, **6**, and **9**. Therefore, it can be inferred that the reduced activity of these compounds against MAO-A may be attributed to the absence of hydrogen bonding interactions. Furthermore, in the active compounds, namely, **11** and **12**, hydrogen bonding and π–π interactions were observed to play a significant role in mediating MAO-A inhibition. Compound **11** forms a strong hydrogen bond with Tyr-444 at the active site and also exhibits π–π interactions with Tyr-407, Gly-110, and Ile-335. The best docking pose of compound **12** involved a hydrogen bond with Gly-110 and π–π interactions with Tyr-407, Ile-180, and Ile-335 of MAO-A.

The tested coumarin derivatives were also found within the catalytic binding pocket of MAO-B. The active site of MAO-B comprises a bipartite cavity that includes the substrate cavity, which is located in front of the FAD, and the entrance cavity, which is positioned beneath the protein surface and enclosed by a loop formed by lipophilic region residues. The binding mode of all coumarins in MAO-B traversed both the binding pockets: the entrance cavity lined by residues Phe-168, Leu-171, Ile-198, Ile-199, and Tyr-326 and the substrate cavity bordered by residues Tyr-60, Trp-119, Leu-164, Leu-167, Cys-172, Gln-206, Tyr-398, and Tyr-435. This arrangement closely resembled the binding sites of the coumarin derivative 7-(3-chlorobenzyloxy)-4-(methylamino)methylcoumarin, which is a reference MAO-B inhibitor (Figure 4A). Additionally, all the coumarins displayed hydrogen-bonding interactions. Hydrogen-bonding interactions with Gln-206, Tyr-435, and Ile-198 were observed for compounds **5**, **10**, **11**, and **12**; interactions with Pro-104, Ile-198, and Leu-171 were observed for compound **3**; interactions with Gly-58, Tyr-326, Pro-102, Arg-42, and Ile-199 were observed for compound **4**; interactions with Gly-58, Cys-172, Lys-296, and Tyr-398 were observed for compound **7**; interactions with Gly-434 and Gln-206 were observed for compound **2**; interactions with Pro-104, Ile199, and Gln-206 were observed for compound **1**; interactions with residue Ile-198 were observed for compounds **8** and **9**; and interactions with residues Leu-171 and Gln-206 were observed for compound **6**. Additionally, all compounds showed π–π interactions, except compounds **8** and **12**. The π–π interactions were observed with Tyr-326 for compounds **1**, **2**, **3**, and **9**; interactions with Tyr-398 were observed for compounds **1**, **2**, **5**, **6**, **9**, **10**, and **11**; interactions with Tyr-435 were observed for compounds **1**, **2**, **3**, and **7**; and interactions with Gly-57 were observed for compounds **4** and **10**. Additionally, π–σ interactions have been observed with Ile-199 for compounds **2**, **3**, **5**, and **11**; interactions with Tyr-398 have been observed for compounds **3** and **4**; and interactions with Gly-58 have been observed for compound **9**. Hence, in the majority of MAO-B active coumarin derivatives, π–π and hydrogen bonding interactions were predominantly observed to mediate MAO-B inhibition.

### 2.4. Biological Profile of Coumarin Derivatives as Potential Drugs for Treatment of AD

#### 2.4.1. Evaluation of hAChE Inhibitory Activity

As shown in Table 1, arylpiperazinyl derivatives of coumarin displayed varied hAChE inhibitory activity compared to the reference compound Ensaculin. The highest activity was found in the 8-acetyl derivatives **10**, **11**, and **12** with the following values: **10** (IC_50_ = 1.52 ± 0.66 μM) > **11** (IC_50_ = 2.80 ± 0.69 μM) > **12** (IC_50_ = 4.95 ± 0.48 μM). Compounds **1**–**9** did not show significant activity, with inhibition values ranging from 46% for compound **7** to 61% for compounds **1** and **2**. The structure–activity studies revealed that the derivatives of 4,7-dimethyl-5-hydroxycoumarin (**1**–**4**) and 6-acetyl-4,7-dimethyl-5-hydroxycoumarin (**5**–**8**) were inactive. Only derivatives of 8-acetyl-7-hydroxy-4-methylcoumarin exhibited hAChE inhibitory activity. Interestingly, 8-acetyl-7-{3-[4-(2-methoxyphenyl)piperazin-1-yl]butoxy}-4-methylchromen-2-one (**10**) showed the highest activity (IC_50_ = 1.52 μM), while its analog 8-acetyl-7-{3-[4-(2-methoxyphenyl)piperazin-1-yl]propoxy}-4-methylchromen-2-one (**9**) was less active (59% of inhibition). These results may suggest that shortening the linker between coumarin and piperazine moiety from four to three carbon atoms resulted in a decrease in activity. This phenomenon occurred only when the methoxy substituent on the phenyl ring of piperazine was in the C2 position. For the 3-methoxyphenyl moiety, both compounds with a three-carbon and four-carbon linkers were active, with IC_50_ values of 2.80 μM for 8-acetyl-7-{3-[4-(3-methoxyphenyl)piperazin-1-yl]propoxy}-4-methylchromen-2-one (**11**) and 4.95 μM for 8-acetyl-7-{4-[4-(3-methoxyphenyl)piperazin-1-yl]butoxy}-4-methylchromen-2-one (**12**). Additionally, compound **11**, with a three-carbon linker, exhibited better hAChE inhibitory activity than compound **12**, with a four-carbon linker.

#### 2.4.2. Evaluation of hMAOs Inhibitory Activity

Compounds **11** and **12** showed a good inhibitor profile against hMAO-A, with IC_50_ values of 6.97 μM and 7.65 μM, respectively, compared to the reference compound, pargyline (IC_50_ = 10.9 μM). The remaining derivatives, **1**–**10,** were inactive. This indicates that in terms of MAO-A inhibitory activity, only derivatives of 8-acetyl-7-hydroxycoumarin exhibited activity, while derivatives of 5-hydroxy and 6-acetyl-5-hydroxycoumarin had no effect. Additionally, the activity was ensured by the substitution of (3-methoxyphenyl)piperazine connected with a three- or four-carbon linker to the coumarin moiety. Replacing (3-methoxyphenyl)piperazine with (2-methoxyphenyl)piperazine resulted in a drastic decrease in the activity of compounds. Consequently, derivatives **9** and **10** with the (2-methoxyphenyl)piperazine moiety were inactive, showing only 32% and 52% inhibition, respectively.

In the case of inhibitory activity against MAO-B, the situation was completely different. The highest activity was found for compounds **1**, **3**, **4**, and **9** (Table 1), with the following values: **3** (IC_50_ = 1.88 μM) > **1** (IC_50_ = 2.18 μM) > **4** (IC_50_ = 3.18 μM) > **9** (IC_50_ = 4.76 μM). The derivatives of 4,7-dimethyl-5-hydroxycoumarin with the three-carbon linker (**3** and **1**) were the most active, with compound **3**, containing a 3-methoxyphenyl moiety, performing the best. The introduction of the 2-methoxyphenyl moiety only slightly affected the activity, changing from IC_50_ = 1.88 μM for compound **3** to IC_50_ = 2.18 μM for compound **1**. Similarly, extending the linker between the coumarin and piperazine moiety from three carbons to four carbons changed the activity from IC_50_ = 1.88 μM for compound **3** to IC_50_ = 3.18 μM for compound **4**. In the group of 8-acetyl-7-hydroxycoumarin derivatives, only 8-acetyl-7-{3-[4-(2-methoxyphenyl)piperazin-1-yl]propoxy}-4-methylchromen-2-one (**9**) showed activity at the level of pargyline (IC_50_ = 4.76 μM for compound **9,** compared to IC_50_ = 2.69 μM for pargyline). In this group, any change in the length of the linker or the position of the methoxy substituent on the phenyl ring resulted in a significant decrease in activity.

### 2.5. Molecular Dynamics Analysis of hAChE Inhibitors

#### 2.5.1. Pose Analysis and Binding Mode of hAChE Inhibitors **10**–**12**

**Figure 5:**
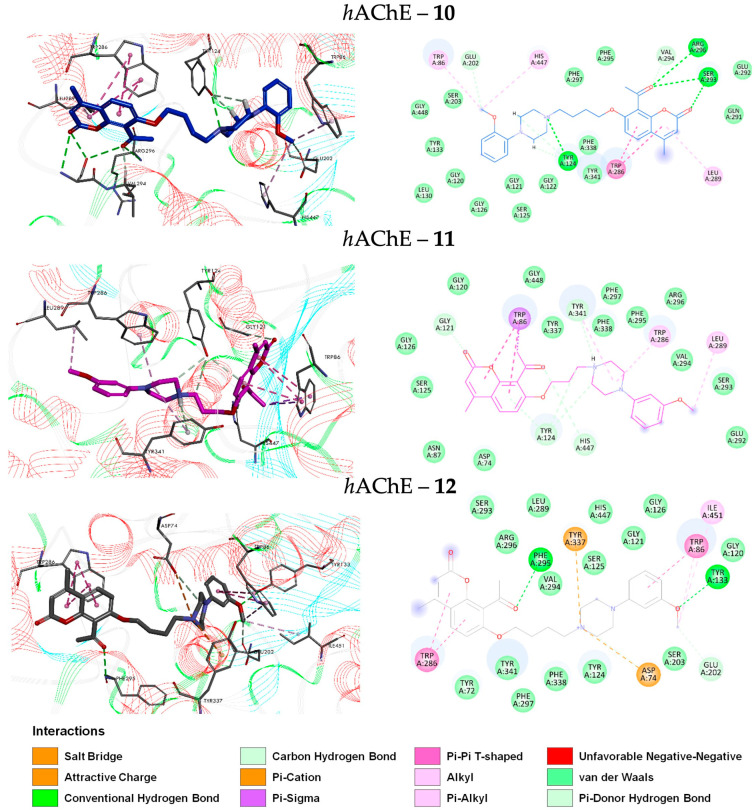
Structures of the **10**–hAChE, **11**–hAChE, and **12**–hAChE complexes and 2D view of all hAChE residues interacting with the ligands resulting from MD simulations (residues involved in hydrogen bonding marked as green and cyan circles; residues involved in hydrophobic interactions marked as pink circles; and electrostatic interactions marked as orange circles).

As indicated by the biological results, 7-*O*-arylpiperazinylcoumarins (**10**, **11**, and **12**) showed selective hAChE inhibitory activity. Molecular dynamics were employed to gain insight into the binding modes of these compounds. The RMSD values between the starting ligand structures and the resulting average structures were consistently low in all cases. The binding free energy values for compounds **10**–**12** that were calculated using the MM-GBSA method are presented in Table 1, together with the experimental values IC_50_ against hAChE. The calculated strength of interactions with hAChE decreases in the sequence **10** > **11** > **12** (−77.24 kcal/mol > −76.32 kcal/mol > −70.12 kcal/mol, respectively), which correlates well with the experimental data. Compound **10**, which exhibited the lowest binding energy (−77.24 kcal/mol), emerged as the most potent hAChE inhibitor among the selected coumarin derivatives, which is consistent with the experimental study (IC_50_ = 1.52 μM). The MD-generated orientations of ligands are presented in Figure 5. Compounds **10** and **12**, with a butylene linker, are predicted to be accommodated in the hAChE pocket in extended conformations, whereas compound **11**, with a propylene linker, adopted a folded conformation. Furthermore, it was observed that the coumarin moiety of compounds **10** and **12** is oriented toward the PAS of the enzyme, whereas in compound **11**, the coumarin moiety is positioned in the CAS region.

The butoxy derivative **10** with the 2-methoxyphenyl substituent, which is one of the most potent hAChE inhibitors among the tested coumarin series, shows favorable interactions within the binding site. It forms hydrogen bonds between the carbonyl group and the acetyl substituent at the 8-position of the coumarin moiety, with the hydrophilic pocket composed of Ser-293, Arg-296, and Val-294 (with lengths of 2.17, 3.23, and 2.33 Å, respectively). Additionally, hydrogen bond interactions were observed between the protonated piperazine moiety and the methoxy group at the 2-position of the phenyl ring, with Tyr-124 in the middle gorge and Glu-202 (with lengths of 2.60, 2.89, 3.01, and 3.24 Å, respectively). Moreover, the coumarin moiety of compound **10** occupies the PAS of hAChE and engages in double π–π stacking interactions with the indole ring of Trp-286 (distances of 4.05 and 5.07 Å) and a π-alkyl interaction with Leu-289 (distance of 4.23 Å). Furthermore, the methoxy group at the 2-position of the phenyl ring forms π interactions with the aromatic ring of Trp-86 (distance of 4.07 Å) and with His-447 (distance of 5.04 Å).

In the case of the less active butoxy derivative **12** with the 3-methoxyphenyl substituent, the coumarin moiety demonstrates double π–π stacking interactions with the indole ring of Trp-286 located in the PAS region of AChE (distances of 3.72 and 5.09 Å). Additionally, an electrostatic interaction is observed between the protonated piperazine and Asp-74 in the PAS, as well as Tyr-337 (with distances of 4.06 and 4.43 Å, respectively). On the other hand, the 3-methoxyphenyl moiety of compound **12** binds to the CAS region, where it engages in a hydrophobic interaction with residues Trp-86 (5.02 Å) and Ile-451 (5.39 Å). Hydrogen bonding interactions are observed between the acetyl group at the 8-position of the coumarin ring and Phe-295 in the middle gorge residue (distance of 3.00 Å), as well as between the methoxy group at the 3-position of the phenyl ring and Tyr-131 in the CAS region (distance of 2.74 Å). Another interesting case is represented by compound **11**, with a propylene linker and a 3-methoxyphenyl substituent. In this compound, there is an inversion of the binding mode compared to compounds **10** and **12**. In compound **11**, the coumarin moiety is bound to the catalytic anionic site (CAS), exhibiting a hydrophobic interaction with the quaternary ammonium binding locus Trp-86 (lengths of 4.84 and 4.92 Å). In addition, the oxygen atom in the carbonyl group and acetyl substituent at the 8-position form hydrogen bonds with the residue Gly-121 (3.30 Å) and His-447 (3.65 Å) in CAS residues, which further enhances the binding ability to this site. Compound **11** creates hydrophobic and hydrogen bond interactions with interior pocket residues Tyr-341, Tyr-124, and Trp-286 involving the protonated piperazine. In addition to these typical interactions, the folded conformation of **11** allows favorable interactions of the 3-methoxyphenyl moiety with Leu-289. Taken together, all these results suggest that compounds **10**, **11**, and **12** could occupy the entire enzyme active sites, acting as dual-binding site inhibitors of hAChE. The higher potency of the compounds with 7-*O*-arylpiperazinylcoumarin could be attributed to more favorable interactions with the target enzyme.

#### 2.5.2. Fundamental Molecular Dynamics Simulation Analysis of hAChE Inhibitors **10**–**12**

**Figure 6:**
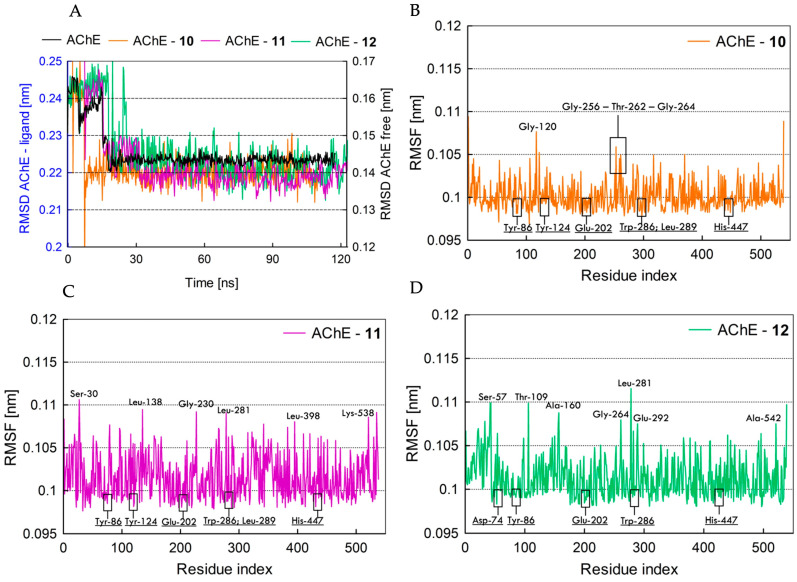
Trajectory analysis of the MD simulation of complexes of AChE with compounds **10**, **11,** and **12**. (**A**) Plots of root mean square deviation (RMSD) and (**B**–**D**) root mean square fluctuation (RMSF) over 120 ns o protein-ligand complexes.

Molecular docking provides a depiction of the enzyme–ligand complex in a stable conformation. However, the ligand continually moves within the enzyme’s active site pocket [40]. Therefore, to observe the motion of ligands in the enzyme’s active site, we performed MD simulations to explore different conformations and assess the stability of the inhibitor–hAChE complexes. From the twelve compounds evaluated, three C7-coumarin derivatives (**10**, **11**, and **12**) were selected for MD simulation analysis based on the agreement of experimental and computational evidence regarding their conformations with the hAChE enzyme. The stability of selected enzyme–ligand complexes was analyzed using root mean square deviation (RMSD) and root mean square fluctuation (RMSF) over a period of 120 ns of simulation (see Figure 6). The RMSD values of the atoms in free hAChE and the hAChE–ligand complexes were plotted over a range of 0–120 ns, as shown in Figure 6A. It can be observed that the RMSD deviation amplitude for compounds **10** and **11** in the complex with hAChE is greater compared to the stability observed in the compound **12**-hAChE complex. A smaller deviation curve indicates higher stability and vice versa. Considerable fluctuations were noted in the RMSD values across all three complexes during the initial 20 ns. However, after 20 ns, the complexes began to stabilize, indicating the compounds’ stable behavior through the strong inhibition of the target and explicit binding to the active site.

Afterward, we analyzed the RMSF to investigate the fluctuations of each residue in the enzyme complexed with three compounds, **10**, **11**, and **12**, during the simulations. The residues found to interact with the ligands exhibited greater stability and fewer fluctuations. Minimal fluctuations were observed in the residues of the binding pocket across all complexes, indicating satisfactory stability of complexes throughout the MD simulation. The RMSF of the protein showed flexibility within a range of 0.097 to 0.115 nm for all three compounds (Figure 6B–D). The active residues of CAS (Trp-86, Glu-202, His-447, and Tyr-124) and PAS (Asp-74, Leu-289, and Trp-286) that bound to ligands showed the lowest fluctuation amplitude in the RMSF plots. The N- and C-terminal regions, which can move freely, showed high fluctuations. The hAChE structure consists of many loops that are also free to move and can undergo conformational changes. The amino acid residues in the protein side chain for compound **10**, specifically, Gly-120, Gly-256, Thr-262, and Gly-264 (Figure 6B); compound **11**, specifically, Ser-30, Leu-138, Gly-230, Leu-281, Leu-398, and Lys-538 (Figure 6C); and compound **12**, specifically, Ser-57, Thr-109, Ala-160, Gly-264, Leu-281, Glu-292, and Ala-542 (Figure 6D), showed fluctuations of more than 0.108 nm due to their positions on the protein’s outer exposed surface. As these fluctuating residues were not present in the binding pocket, they did not impact the ligand binding stability.

### 2.6. Molecular Dynamics Analysis of hMAOs Inhibitors

#### 2.6.1. Pose Analysis and Binding Mode of hMAO-A Inhibitors **11** and **12**

**Figure 7:**
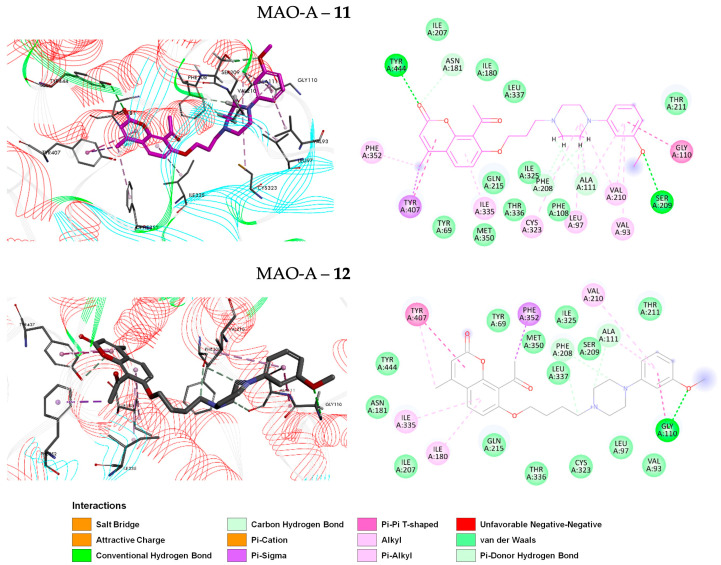
Structures of the **11**–hMAO-A and **12**–hMAO-A complexes and 2D view of all hMAO-A residues interacting with the ligands resulting from MD simulations (residues involved in hydrogen bonding marked as green and cyan circles; residues involved in hydrophobic interactions marked as pink circles; and electrostatic interactions marked as orange circles).

Biological results indicate that 7-O-arylpiperazinylcoumarins (compounds **11** and **12**) exhibited selective inhibitory effects not only on hAChE but also on hMAO-A. To gain further insight into their selective hMAO-A inhibitory activity, the binding modes of compounds **11** and **12** in hMAO-A were examined using molecular dynamics. To assess the conformational stability of the complex based on its binding energy, the binding free energy was calculated using the MM-GBSA method. The binding affinities decrease in the sequence **11** > **12** (−66.27 kcal/mol > −62.89 kcal/mol), which correlates with the experimental study (Table 1). Compound **11**, with the propylene linker and the 3-methoxyphenyl substituent, is a potent hMAO-A inhibitor, with an IC_50_ value of 6.67 ± 0.76 μM. It is approximately 1.2-fold more potent than compound **12** (8-acetyl-7-{4-[4-(3-methoxyphenyl)piperazin-1-yl]butoxy}-4-methylchromen-2-one), which shows an IC_50_ of 7.65 ± 0.32 μM. The MD resulting orientations of the average structures of hMAO-A-ligand complexes are depicted in Figure 7.

Compounds **11** and **12** were observed to bind to the active site of hMAO-A primarily through hydrophobic interactions (π–π stacking, π–alkyl, and π–sigma), supplemented by several hydrogen bonds. The coumarin rings of both compounds were similarly located within the hydrophobic binding pocket of hMAO-A, interacting with the side chains of Ile-180, Ile-335, and Tyr-407. Additionally, the acetyl group at the 8-position in compound **12** and the methyl group at the 4-position in compound **11** form π–sigma interactions with Phe-352 (at distances of 3.94 Å and 3.90 Å, respectively). The carbonyl group of the coumarin ring in compound **11** formed strong hydrogen bonds with Tyr-444 (2.39 Å) and Asn-181 (2.64 Å). Compound **11** with a propylene linker is predicted to be accommodated in the MAO-A pocket in an extended conformation, as is compound **12** with a butylene linker. The piperazine moiety of both compounds exhibited hydrogen-bonding interactions with the residues Ala-111 and Phe-208, with distances ranging from 2.41 to 3.00 Å. Additional π–alkyl interactions were observed between the piperazine moiety of compound **11** and Leu-97, Cys-323, Ala-111, and Val-210, with lengths of 4.51, 5.26, 4.40, and 3.94 Å, respectively. Moreover, the phenyl substituent of both compounds forms π–π stacking and π–alkyl interactions with residues Ala-111, Val-210, and Gly-110. The methoxy group at the 3-position of the phenyl ring forms a stronger hydrogen bond with Ser-209 (2.09 Å) in compound **11** compared to compound **12**, which interacts with residue Gly-110 (2.46 Å).

#### 2.6.2. Pose Analysis and Binding Mode of hMAO-B Inhibitor **1**, **3** and **4**

**Figure 8:**
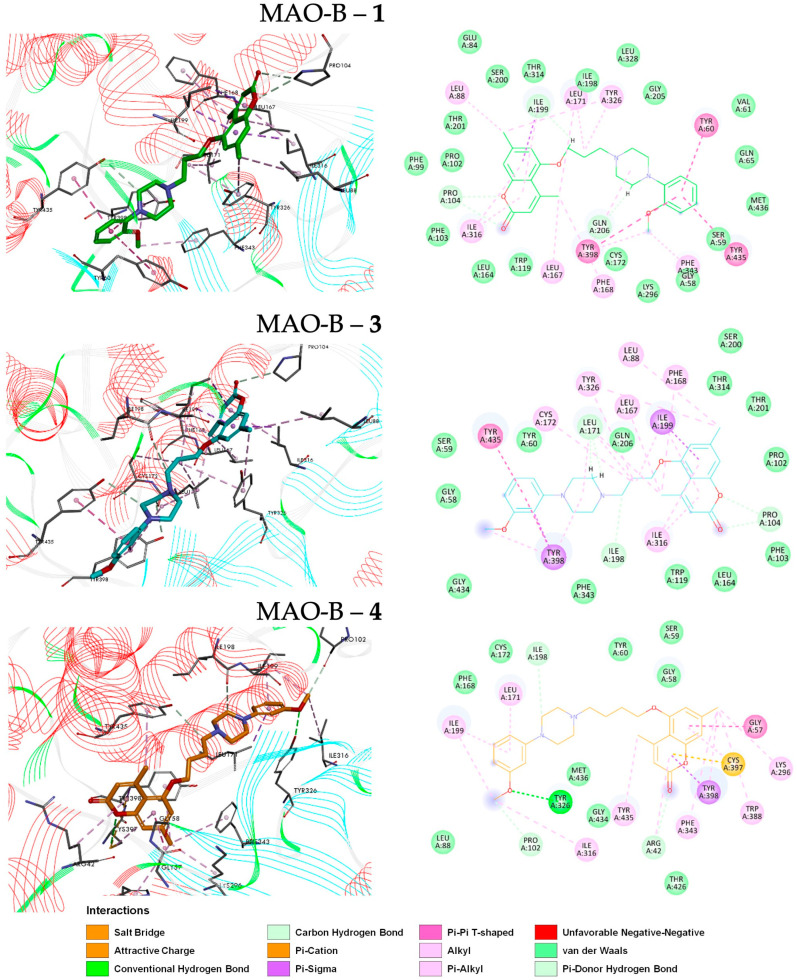
Structures of the **1**–MAO-B, **3**–MAO-B, and **4**–MAO-B complexes and 2D view of all MAO-B residues interacting with the ligands resulting from MD simulations (residues involved in hydrogen bonding marked as green and cyan circles; residues involved in hydrophobic interactions marked as pink circles; and electrostatic interactions marked as orange circles).

A series of 5-*O*-arylpiperazinylcoumarin and 7-*O*-arylpiperazinylcoumarin derivatives were evaluated as monoamine oxidase B inhibitors. The results indicated that primarily 4,7-dimethyl-5-*O*-arylpiperazinylcoumarins selectively inhibited MAO-B, with IC_50_ values ranging from 1.88 to 3.18 µM. Among these, compounds **1** (IC_50_ = 2.18 μM), **3** (IC_50_ = 1.88 μM), and **4** (IC_50_ = 3.18 μM) exhibited the most potent inhibitory activity and the highest selectivity for MAO-B. In addition, the binding modes of these active coumarin derivatives were investigated using MD simulations to explore their interactions within the active site of the hMAO-B complex structure. The MD complexes were subsequently analyzed post-simulation using MM-GBSA to estimate their binding free energies. As noted in Table 1, among the studied protein–ligand complexes, the C5-substituted coumarin derivatives, specifically compounds **1**, **3**, and **4**, in complex with MAO-B exhibited the most negative binding free energy values, indicating stronger binding affinities, which correlates well with the experimental data. The binding affinities decrease in the following sequence: **3** > **1** > **4** (−75.57 kcal/mol > −72.32 kcal/mol > −70.04 kcal/mol). Figure 8 illustrates the intermolecular interactions of compounds **1**, **3,** and **4** with the binding site residues of the MAO-B enzyme.

By analyzing the interactions of compounds **1**, **3,** and **4** with the MAO-B binding site, it can be observed that the coumarin moiety of compound **4**, which has a butylene linker, unlike compounds **1** and **3** with propylene linkers, is located in the substrate cavity. This positioning places the lactone ring close to the FAD cofactor site, where it is stabilized by hydrophobic and electrostatic interactions. Specifically, interactions occur with Tyr-398 (2.86 Å), Cys-397 (4.88 Å), which links to the FAD, and Arg-42 (5.21 Å). Furthermore, the carbonyl oxygen of the coumarin moiety forms a hydrogen bond with Arg-42 (2.78 Å), while the methyl group at the 4- and 7-positions of the coumarin ring engage in π interactions with Lys-296 (4.17 Å), Phe-343 (5.45 Å), Trp-388 (4.79 Å), and Tyr-435 (4.66 Å). These interactions contribute to the stable binding of compound **4** within the MAO-B active site.

The 3-methoxyphenyl moiety of compound **4** occupied the entrance cavity of MAO-B and interacts with several residues through hydrogen bonds, van der Waals forces, and hydrophobic interactions. These interactions occur with Pro-102, Leu-171, Ile-199, Ile-316, and Tyr-326, with distances ranging from 2.25 to 5.11 Å. These interactions collectively contribute to the stabilization and binding of compound **4** within the MAO-B enzyme’s entrance cavity.

The binding mode of compounds **1** and **3** with a propylene linker positions the ligands within the central region of MAO-B’s active site, which is known for effective inhibitor binding. Specifically, compound **3** with the 3-methoxyphenyl moiety shows a similar energetically favorable orientation as compound **1**, which has a 2-methoxyphenyl moiety positioned in the binding pocket. This orientation suggests that both compounds utilize the same binding interactions within the MAO-B active site, contributing to their inhibitory activity. Based on Figure 8, it is evident that the ligands completely occupy the protein binding site. Specifically, the coumarin moiety of the ligands occupies the large lipophilic cavity that forms the entrance cleft of the site. Meanwhile, the (2- or 3-methoxyphenyl)piperazine moiety of the compounds is positioned within the substrate pocket and the region between these two cavities. This arrangement aligns well with the predictions from the docking calculations, highlighting how these compounds interact within the MAO-B binding site to exert their inhibitory effects. The coumarin ring of both compounds forms π–alkyl and π–sigma interactions with residues Ile-199 and Ile-316 and hydrogen bonds with Pro-104 (in the range from 2.49 to 2.63 Å). Additionally, the methyl groups at the 4- and 7-positions form alkyl and π–alkyl interactions with Leu-88, Leu-167, Phe-168, Leu-171, and Tyr-326, with distances ranging from 4.14 to 5.47 Å. In compound **3**, the arylpiperazinyl chain is further stabilized by hydrogen bonds with Ile-198 (2.56 Å), Leu-171 (2.67 and 2.76 Å), and Gln-206 (2.37 Å). It also engages in π–alkyl interactions with Ile-199 (5.41 Å), Tyr-326 (5.35 Å), Cys-172 (5.27 Å), Leu-171 (4.12 and 4.99 Å), and Tyr-398 (5.19 Å). These interactions contribute to a higher affinity compared to compound **1**, enhancing its binding to the MAO-B active site. Moreover, the folded conformation of compound **3** allowed for favorable interactions of the 3-methoxyphenyl substituents with Tyr-398, Tyr-435, and Gly-434. Based on theoretical results, it can be concluded that all structural features, including the substituents and the length of the alkyl chain, play an important role in binding to MAO-B.

### 2.7. Fundamental Molecular Dynamics Simulation Analysis of MAO’s Inhibitors

To investigate the binding affinity to MAO enzymes and secondary structural changes induced by coumarin derivatives, MD simulation trajectories of free MAO-B and the MAO-B–ligand complexes were performed and compared. Structural properties were analyzed using RMSD and RMSF. Based on the agreement of experimental and computational evidence, the conformations of three complexes (MAO-B–**1**, MAO-B–**3**, and MAO-B–**4**) with the lowest binding free energies were chosen. MD simulations were conducted for 120 ns to analyze the conformational stability of these selected complexes.

**Figure 9:**
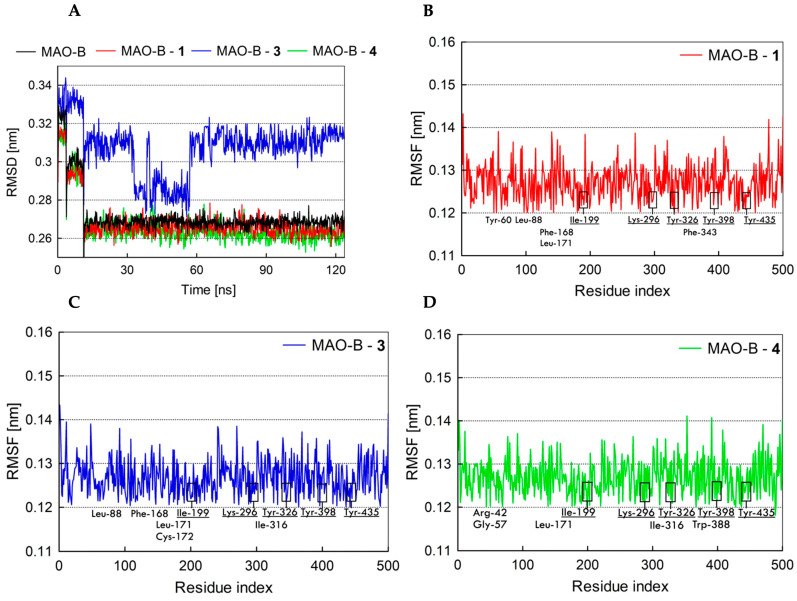
Trajectory analysis of the MD simulation of complexes of MAO-B with compounds **1**, **3,** and **4**. (**A**) Plots of root mean square deviation (RMSD) and (**B**–**D**) root mean square fluctuation (RMSF) over 120 ns o protein-ligand complexes.

The binding modes of the compounds were analyzed in terms of the RMSD deviations of the ligands throughout the simulation time from the starting input structure. The RMSD values of the atoms in free MAO-B and the complexed forms were plotted over the range of 0–120 ns, as shown in Figure 9A. According to the graph, both the free protein and the complexes remained stable, as evidenced by the well-maintained protein conformation and ligand binding mode throughout the MD simulation. During the initial 15 ns of the simulation, there was an increase in the RMSD of the protein backbone, which can be attributed to the removal of the position restraints on the protein α-carbons. The analysis indicated that the RMSD of free MAO-B and complexes (MAO-B–**1** and MAO-B–**4**) reaches equilibrium and oscillates around an average value of approximately 0.27 nm after 15 ns of simulation time. Meanwhile, the MAO-B–**3** complex initially exhibited fluctuations and stabilized after 70 ns of simulation trajectories, with RMSD values stabilizing around 0.31 nm. These results suggest that the conformations of the tested compounds in complex with MAO-B varied at different stages of the MD simulation but ultimately stabilized. This stability observed in the RMSD values aligns with the initial predictions from molecular docking studies.

To understand the mobility of the MAO-B enzyme in the presence of compounds **1**, **3**, and **4** within its binding site, RMSF analysis is crucial. The RMSF provides insights into the flexibility and movement of specific amino acids, which is illustrated in Figure 9B–D. A decrease in the RMSF value typically indicates reduced mobility and higher stability of the residue. This reduction can suggest stronger protein–ligand interactions, particularly for residues near the active site or those involved in interactions with the ligands. It is conceivable that a stronger protein–ligand interaction occurs when the RMSF value is low for residues located near the active site or those involved in the interactions between the ligand and the receptor protein [41,42].

The fluctuation in the C-terminal (membrane binding region) and N-terminal regions was found to be high (above 0.14 nm) compared to other regions of the MAO-B enzyme, likely due to their hydrophobic nature. As shown in Figure 9B–D, for the complexes of compounds **1**, **3**, and **4** with protein, fluctuations ranging from 0.12 to 0.14 nm were observed in the RMSF for each residue surrounding the ligand. This stability indicates that the binding pocket remained stable throughout the MD simulation. Apart from this, the RMSF values of catalytic residues such as Lys-296, orient keeper residues such as Tyr-326, Tyr-398, and Tyr-435, and gate residues such as Ile-199 were found to be very low, suggesting strong intermolecular interactions with the compounds **1**, **3**, and **4** in the ligand–MAO-B complex.

## 3. Materials and Methods

### 3.1. Experimental Section

#### 3.1.1. Chemical Compounds

The starting materials were obtained from Aldrich or Merck and used as received. The reaction processes were conducted using a Plazmatronika-Poland 1000 microwave oven (Wrocław, Poland), and the melting points were measured with an ElectroThermal 9001 Digital Melting Point apparatus (Chelmsford, UK), with values uncorrected. High-resolution mass spectra were obtained using a Shimadzu LCMS-9030 spectrometer (Kyoto, Japan). ^1^H NMR and ^13^C NMR spectra in solution were recorded at 25 °C with a Bruker Advance III HD 300 MHz spectrometer (Karlsruhe, Germany) employing standard Topspin v. 3.2 software. Chemical shifts δ [ppm] were referenced to TMS. TLC was conducted on Kieselgel 60 F254 plates (Sigma-Aldrich, Oakville, ON, Canada), and the spots were detected under UV light at wavelengths of 254 and 365 nm.

#### 3.1.2. General Procedure for the Preparation of Compounds **1**–**12**

The synthesis processes for new compounds **1** and **2** involved two steps. In the first step, a mixture of 5-hydroxy-4,7-dimethylcoumarin (0.190 g, 1 mmol), 1,3-dibrompropane or 1,4-dibromobutane (0.34 cm^3^, 3 mmol of 1,3-dibrompropane for compound **1**; 0.36 cm^3^, 3 mmol of 1,4-dibromobutane for compound **2**), anhydrous K_2_CO_3_ (0.1 g), and a catalytic amount of KI was placed in a microwave flask. The mixture was refluxed in 5 cm^3^ of acetonitrile at 70–80 °C in the monomode microwave oven (300W) for 2 cycles of 6 min each (total heating time: 12 min). The progress of the reaction was monitored by TLC on silica gel plates (eluent/CHCl_3_–MeOH: 10:0.25). After completing the reaction, the solvent was evaporated, and the residue was purified using column chromatography (chloroform/hexane: 5:3). The obtained intermediate products were used for the second step of synthesis; a mixture of 5-(3-bromopropoxy)-4,7-dimethylcoumarin or 5-(3-bromobuthoxy)-4,7-dimethylcoumarin (1 mmol), corresponding amine (2 mmol), anhydrous K_2_CO_3_ (0.3 g), and a catalytic amount of KI were placed in a microwave flask. The mixture was refluxed in 5 cm^3^ of acetonitrile at 70–80 °C in the monomode microwave oven (300W) for 4 cycles of 6 min each (total heating time: 24 min). The progress of the reaction was monitored as in the first step. The mixture was then filtered, the solvent was evaporated, and the residue was purified using column chromatography.

1. 4,7-dimethyl-5-{3-[4-(2-methoxyphenyl)piperazin-1-yl]propoxy}coumarin (**1**)

White solid, MP: 148– 149 °C; yield 65%, ^1^H NMR (300 MHz, CDCl_3_): δ = 6.99 (4H, m, H-2″-H-6″), 6.76 (1H, s H-8), 6.56 (1H, s, H-6), 6.06 (1H, s, H-3), 4.13 (2H, t, *J* = 7.5 Hz, H-1′), 3.89 (1H, s, H-7″), 3.17 (4H, br. s., H-3p, H-5p), 2.77 (4H, br. s., H-2p, H-6p), 2.70 (2H, t, *J* = 7.5 Hz, H-3′), 2.60 (3H, s, H-9), 2.40 (3H, s, H-10), 2.15 (2H, m, H-2′); ^13^C NMR (75 MHz, CDCl_3_): δ = 161.2 (C-1″), 157.3 (C-2), 155.5 (C-3), 154.2 (C-4), 152.4 (C-8a), 143.2 (C-2″), 141.1 (C-7), 123.2 (C-6″), 121.2 (C-4″), 118.4 (C-5″), 113.6 (C-4a), 112.3 (C-3″), 110.4 (C-6), 108.3 (C-3), 108.1 (C-8), 67.4 (C-1′), 55.6 (C-3′), 55.5 (C-3p, C-5p), 53.7 (C-2p, C-6p), 50.5 (C-7″), 26.6 (C-2′), 24.8 (C-9), 22.2 (C-10); TOF MS ES+: [M+H]^+^ calcd for C_25_H_30_O_4_N_2_ (423.2278) found 423.2294.

2. 4,7-dimethyl-5-{4-[4-(2-methoxyphenyl)piperazin-1-yl]buthoxy}coumarin (**2**)

White solid, MP: 131–133 °C; yield 59%, ^1^H NMR (300 MHz, CDCl_3_): δ = 6.93 (4H, m, H-2″-H-6″), 6.75 (1H, s H-8), 6.53 (1H, s, H-6), 6.05 (1H, s, H-3), 4.08 (2H, t, *J* = 7.5 Hz, H-1′), 3.88 (1H, s, H-7″), 3.15 (4H, br. s., H-3p, H-5p), 2.73 (4H, br. s., H-2p, H-6p), 2.60 (3H, s, H-9), 2.55 (2H, t, *J* = 7.5 Hz, H-4′), 2.40 (3H, s, H-10), 1.94 (2H, m, H-2′), 1.78 (2H, m, H-3′); ^13^C NMR (75 MHz, CDCl_3_): δ = 161.2 (C-1″), 157.4 (C-2), 155.5 (C-4), 154.3 (C-5), 152.4 (C-8a), 143.2 (C-2″), 141.2 (C-7), 123.2 (C-6″), 121.1 (C-4″), 118.4 (C-5″), 113.6 (C-4a), 111.3 (C-3″), 110.3 (C-6), 108.3 (C-3), 108.0 (C-8), 68.9 (C-1′), 58.4 (C-4′), 55.5 (C-3p, C-5p), 53.6 (C-4′), 50.6 (C-2p, C-6p), 27.4 (C-2′), 24.8 (C-3′), 23.6 (C-9), 22.2 (C-10); TOF MS ES+: [M+H]^+^ calcd for C_26_H_32_O_4_N_2_ (459.2254) found 459.2276.

The synthesis of the remaining derivatives, **3**–**12,** was carried out analogously, also in two steps. For derivatives **3** and **4**, the starting compound in the first step was 5-hydroxy-4,7-dimethylcoumarin (1 mmol) combined with 1,3-dibromopropane or 1,4-dibromobutane (3 mmol), respectively. In the second step, 3-methoxyphenylpiperazine (2 mmol) was used for both compounds **3** and **4**. For derivatives **5**–**8**, the starting substrate was 6-acetyl-5-hydroxy-4,7-dimethylcoumarin (1 mmol). In the first step, 1,3-dibromopropane (3 mmol) was used for compounds **5** and **7**, while 1,4-dibromobutane (3 mmol) was used for compounds **6** and **8**. In the second step, 2-methoxyphenylpiperazine (2 mmol) was employed to obtain derivatives **5** and **6**, while 3-methoxyphenylpiperazine (2 mmol) was used to obtain derivatives **7** and **8**. To obtain derivatives **9**–**12**, 8-acetyl-7-hydroxy-4-methylcoumarin (1 mmol) was used as the starting compound in the first step, along with either 1,3-dibromopropane (3 mmol) for compounds **9** and **11** or 1,4-dibromobutane (3 mmol) for compounds **10** and **12**. In the second step, 2-methoxyphenylpiperazine (2 mmol) was used to synthesize derivatives **9** and **10**, while 3-methoxyphenylpiperazine (2 mmol) was employed to obtain derivatives **11** and **12**. ^1^H NMR and ^13^C NMR spectra for compounds **3**–**12** are available in the ESI; spectra for compounds **3**–**4** and **7**–**8** are available in [35]; spectra for compounds **5**–**6** are available in [36]; spectra for compounds **9**–**10** are available in [34]; and spectra for compounds **11**–**12** are available in [33].

### 3.2. Biological Assays

All enzymes and chemicals were purchased from Aldrich (Milan, Italy). Inhibition assays were performed in 96-well plates (Greiner Bio-One GmbH, Frickenhausen, Germany) using an Infinite M1000 plate reader (Tecan, Milan, Italy). Incubations were performed in triplicate, and final inhibition values were determined as mean ± SEM (n = 3). IC_50_ values were obtained from nonlinear regression using Prism software (version 5.01 for Windows; GraphPad Software, San Diego, CA, USA).

#### 3.2.1. In Vitro Inhibition Studies on hAChE

The inhibition of human recombinant AChE was determined as already reported [43] by applying Ellman’s spectrophotometric method. Briefly, the enzyme was incubated at 25 °C with an inhibitor (10 μM for single-point assay and 7 concentrations ranging from 30 to 0.01 μM for IC_50_ determinations) and chromophoric reagent 5,5-dithio-*bis*-(2-nitrobenzoic acid) in phosphate buffer with a pH of 8.0. After the addition of substrate acetylthiocholine, the increase in absorbance was read at 412 nm for 5 min and compared with a control sample devoid of inhibitor.

#### 3.2.2. In Vitro Inhibition Studies on hMAOs

The inhibition of hMAO-A and B isoforms was determined using a reported spectrofluorimetric procedure [44] by reading the fluorescence (ex. 310, em. 400 nm) of 4-hydroxyquinoline as the product of oxidative deamination of substrate kynuramine. Incubations were set at 37 °C in phosphate buffer with a pH of 7.4 for 20 min before the addition of enzyme, and the fluorescence was measured after additional incubation of 30 min.

### 3.3. Theoretical Methodology

#### 3.3.1. Preparation of the Target Biomacromolecules

The crystallographic structures of human MAO-A, MAO-B, and AChE were retrieved from the RCSB Protein Data Bank (https://www.rcsb.org, accessed on 20 January 2024). The structure of MAO-A with the highest resolution (PDB ID: 2Z5Y, resolution = 2.1 Å), bound to the known inhibitor harmine was selected [38]. The MAO-B structure bound to a coumarin derivative (7-(3-chlorobenzyloxy)-4-(methylamino)methylcoumarin) with the highest resolution was chosen (PDB ID: 2V61, resolution = 1.7 Å). The co-crystallized coumarin scaffold in this structure was used to superimpose docked ligands and select the binding pose most similar to that of the native ligand [45]. The X-ray crystal structure of the human AChE was selected in a complex with donepezil (PDB ID: 4EY7 (chain A), resolution = 2.35 Å). Co-crystalized ligands and water molecules were removed, as they were not involved in the ligand binding. This deletion facilitated computations and cleared the binding pocket of potential water molecules that could distort the pose search [46]. The flavin adenine dinucleotide (FAD) cofactor was retained because it plays a crucial role in the enzyme’s proper functioning in catalyzing the deamination of monoamines and in investigating its involvement in ligand binding to the enzyme during the molecular docking study [47]. The enzyme preparation wizard in Schrödinger Maestro v.13.3 software was used to assign bond orders, add distinct hydrogens to the structure, and fix any missing atoms in the side chains using Prime [48,49]. The protonation state of ionizable enzyme side chains was assigned at pH = 7. The bound native ligands were used to define the active site residues of the target enzymes. Subsequently, the enzymes were optimized, and their energy was minimized using the OPLS4 force field.

#### 3.3.2. Preparation of Ligands

Twelve compounds were selected and studied (Figure 1): 4,7-dimethyl-5-[3-(4-(2-methoxyphenyl)piperazin-1-yl)propoxy]coumarin) (**1**), 4,7-dimethyl-5-[4-(4-(2-methoxyphenyl)piperazin-1-yl)butoxy]coumarin (**2**), 4,7-dimethyl-5-[3-(4-(3-methoxyphenyl)piperazin-1-yl)propoxy]coumarin) (**3**), 4,7-dimethyl-5-[4-(4-(3-methoxyphenyl)piperazin-1-yl)butoxy]coumarin (**4**), 6-acetyl-4,7-dimethyl-5-[3-(4-(2-methoxyphenyl)piperazin-1-yl)propoxy]coumarin (**5**), 6-acetyl-4,7-dimethyl-5-[4-(4-(2-methoxyphenyl)piperazin-1-yl)butoxy]coumarin (**6**), 6-acetyl-4,7-dimethyl-5-[3-(4-(3-methoxyphenyl)piperazin-1-yl)propoxy]coumarin (**7**), 6-acetyl-4,7-dimethyl-5-[4-(4-(3-methoxyphenyl)piperazin-1-yl)butoxy]coumarin (**8**), 8-acetyl-7-{3-[4-(2-methoxyphenyl)piperazin-1-yl]propoxy}-4-methylchromen-2-one) (**9**), 8-acetyl-7-{4-[4-(2-methoxyphenyl)piperazin-1-yl]butoxy}-4-methylchromen-2-one) (**10**), 8-acetyl-7-{3-[4-(3-methoxyphenyl)piperazin-1-yl]propoxy}-4-methylchromen-2-one) (**11**), and 8-acetyl-7-{4-[4-(3-methoxyphenyl)piperazin-1-yl]butoxy}-4-methylchromen-2-one) (**12**). They belong to two groups of coumarin derivatives, which differ in the length of the linker between the coumarin and the piperidine rings: compounds **1**, **3**, **5**, **7**, **9**, and **11** have propylene chains, and compounds **2**, **4**, **6**, **8**, **10,** and **12** have butylene chains. The starting structures of the coumarin derivatives were constructed using Maestro v. 13.3 software. The geometries of all compounds were optimized using the density functional theory (DFT) with B3LYP/6-311G hybrid functional, as implemented in Gaussian 16 [50]. ESP-atomic partial charges on all atoms were computed using the Breneman model, which reproduces the molecular electrostatic potential [51].

#### 3.3.3. Molecular Structures and Initial System Configurations

Molecular docking was employed to analyze the interactions between the coumarin derivatives and the active sites of hAChE, hMAO-A, and hMAO-B. Ligand docking was performed using the Glide program in Maestro v. 13.3 [48]. The co-crystalized ligands were used to define the grid box placement, with a spacing of 1 Å, utilizing the receptor grid generation tool of Maestro 13.3. The grid dimensions were set to sufficiently encompass the active site residues in all three proteins, with dimensions of 24 × 24 × 24 Å in the x, y, and z directions, respectively. The active site residues of the proteins—the catalytic binding sites (CASs)—include the catalytic triad Ser-203, Glu-334, and His-447, along with PAS residues Tyr-72, Asp-74, Tyr-124, and Trp-286 for hAChE; Tyr-69, Asn-181, Phe-208, Val-210, Gln-215, Cys-323, Ile-325, Ile-335, Leu-337, Phe-352, Tyr-407, and Tyr-444 for hMAO-A; and Tyr-60, Pro-102, Pro-104, Leu-164, Phe-168, Leu-171, Cys-172, Ile-198, Ile-199, Gln-206, Ile-316, Tyr-326, Phe-343, Tyr-398, and Tyr-435 for hMAO-B.. Docked ligand conformations were selected based on their binding energy and their structural similarity to the native ligands. The conformers with the lowest free energy were subsequently used as starting points for the molecular dynamics (MD) simulations.

#### 3.3.4. Molecular Dynamics Simulations and Binding Free Energy Calculations

MD simulations of the coumarin derivative–enzyme complexes were conducted in solution to better replicate experimental conditions and to capture the intermolecular interactions crucial for their stability. Since the FAD cofactor did not exhibit any interactions with the coumarin derivatives during molecular docking studies with hMAO-A and hMAO-B, it was excluded from the MD simulation analysis. Missing side chains and loops in the enzyme structures were reconstructed using the Prime tool. The MD simulations were carried out using the Desmond module to examine structural changes in the enzymes within a solvent environment [48]. The solvated system, employing TIP3P water molecules, was constructed in Desmond using the System Builder module. For the simulations, the complex was centered within an orthorhombic cubic box with periodic boundary conditions. The box was filled with single-point charge water molecules, ensuring a minimum buffer distance of 10 Å between any enzyme atom and the box edges [52]. The system was neutralized by randomly adding an appropriate number of Na^+^ and Cl^−^ counter-ions, and an isosmotic state was maintained by adding 0.15 M NaCl. Subsequently, the solvated system was energy-minimized and relaxed using the OPLS4 force field parameters, following the default protocol provided by the Desmond module [53]. A constant temperature of 300 K and a pressure of 1 atm were maintained throughout the simulation using the Nosé–Hoover thermostat and the Martyna–Tobias–Klein barostat algorithms [54]. The NPT ensemble, with a temperature of 300 K and a pressure of 1 bar, was applied for all simulations. Each run lasted 120 ns, with a relaxation time of 1 ps for the ligands. Binding free energy calculations were subsequently performed using the Prime MM-GBSA approach, employing the variable dielectric surface generalized born (VSGB) model as the implicit solvent and the OPLS4 force field for the resulting enzyme–ligand complexes, to estimate ligand binding affinities [55]. The values were calculated based on the following equation [56]: Δ*G*_bind_ = Δ*E* + Δ*G*_solv_ + Δ*G*_SA_, while Δ*E* = *E*_complex_ − *E*_enzyme_ − *E*_ligand_ where *E*_complex_, *E*_enzyme_, and *E*_ligand_ are the minimized energies of enzyme–ligand complex, enzyme, and ligand, respectively. Δ*G*_solv_ represents the difference in the GBSA solvation energy between the enzyme–ligand complex and the sum of the solvation energies of the enzyme and ligand in the unbound state. Δ*G*_SA_ corresponds to the difference in surface area energies between the complex and the sum of the surface area energies of the free enzyme and ligand. Parameters such as root mean square deviation (RMSD), root mean square fluctuation (RMSF), and enzyme–ligand interactions were analyzed to assess the stability of the enzyme–ligand complexes.

## 4. Conclusions

Drug discovery in Alzheimer’s disease is shifting from single-target modulation to a multi-target-directed ligand strategy. To develop effective drugs for the treatment of AD, a series of new multifunctional hybrids (**1**–**12**) combining coumarin with an arylpiperazinyl moiety were designed and evaluated using a combination of experimental and theoretical methodologies. Arylpiperazinyl derivatives of coumarin exhibited varied hAChE inhibitory activity compared to the reference compound, Ensaculin. The results showed that most of these compounds exhibited high inhibitory activity and selectivity for hAChE and hMAO-B compared to hMAO-A. The biological results showed that compound 8-acetyl-7-{4-[4-(2-methoxyphenyl)piperazin-1-yl]butoxy}-4-methylchromen-2-one (10) (IC_50_ = 1.52 μM) was the most potent derivative for hAChE, while compounds 8-acetyl-7-{3-[4-(3-methoxyphenyl)piperazin-1-yl]propoxy}-4-methylchromen-2-one (**11**) and 4,7-dimethyl-5-[3-(4-(3-methoxyphenyl)piperazin-1-yl)propoxy]coumarin (**3**) exhibited the highest inhibitory activity against hMAOs (IC_50_ = 6.97 μM for hMAO-A—**11** and IC_50_ = 1.88 μM for hMAO-B—**3**). Our results confirmed the peculiar feature of coumarin substitution patterns, particularly that 8-substitution is detrimental to MAO-B activity while leading to selective MAO-A inhibitors [57]. Among these coumarin derivatives, compounds **11** and **12** exhibited potent and balanced inhibitory activity against both hAChE and hMAO-A. Enzyme inhibition data and molecular modeling studies revealed that these compounds could bind simultaneously to the PAS and CAS of hAChE as well as occupy the substrate binding site in hMAO. The simulation analyses of these inhibitors suggest specific prerequisites for the rational or intuitive design of inhibitors. The nature and length of the linkers connecting the arylpiperazinyl moiety and coumarin fragments were found to play a significant role in determining the levels of hAChE and hMAO inhibition. Additionally, the size and position of substituents on both the coumarin and arylpiperazinyl moiety were critical factors. Based on in silico and in vitro studies, compounds **3** and **10** have been found to exhibit excellent pharmacokinetic properties [58,59] and strong AChE/MAO-B inhibitory activity, making them promising candidates for further investigation. Their structures could serve as new scaffolds for designing innovative multifunctional drugs for the treatment of AD disease.

## Figures and Tables

**Table 1:** Inhibitory activity against hAChE, hMAO-A, and hMAO-B and MM-GBSA binding free energy for compounds **1**–**12**.

Compounds	hAChE ^[a,b]^	hMAO-A ^[a,b]^	hMAO-B ^[a,b]^	Δ*G*_bind_ [kcal/mol]		
				hAChE	hMAO-A	hMAO-B
**1**	61 ± 1%	44 ± 4%	**2.18 ± 0.48**	−66.89	−52.88	−72.32
**2**	61 ± 4%	19 ± 1%	33 ± 2%	−66.30	−9.56	−53.61
**3**	53 ± 2%	39 ± 5%	**1.88 ± 0.45**	−65.30	−26.74	−75.57
**4**	56 ± 1%	38 ± 4%	**3.18 ± 0.63**	−65.78	−24.47	−70.04
**5**	50 ± 6%	22 ± 3%	34 ± 2%	−61.09	−10.86	−45.51
**6**	48 ± 1%	30 ± 5%	43 ± 3%	−60.44	−14.87	−58.17
**7**	46 ± 3%	39 ± 3%	35 ± 3%	−60.42	−25.85	−45.07
**8**	54 ± 8%	55 ± 2%	26 ± 2%	−62.24	−59.35	−50.82
**9**	59 ± 7%	32 ± 4%	**4.76 ± 0.34**	−66.25	−17.42	−69.95
**10**	**1.52 ± 0.66**	52 ± 4%	24 ± 1%	−77.24	−56.26	−59.12
**11**	**2.80 ± 0.69**	**6.97 ± 0.76**	32 ± 1%	−76.32	−66.27	−55.07
**12**	**4.95 ± 0.48**	**7.65 ± 0.36**	57 ± 4%	−70.12	−62.89	−50.82
Ensaculin	0.36 ± 0.01	-	-			
Pargyline	-	10.9 ± 0.6	2.69 ± 0.48			

^[a]^ Values are the means of three independent experiments and are expressed as IC_50_ ± SEM ([μM], in bold) or % inhibition at 10 μM ± SD. ^[b]^ Human recombinant AChE and MAOs.

## Data Availability

The data that support the findings of this study are available within the article, its Supplementary Materials, and from the corresponding author, T.Ż.

## Supplementary Materials

The supporting information can be downloaded at https://www.mdpi.com/article/10.3390/ijms252312803/s1.
