# Supplementary material for: Coumarin Derivative Hybrids: Novel Dual Inhibitors Targeting Acetylcholinesterase and Monoamine Oxidases for Alzheimer’s Therapy
Source: Int J Mol Sci. 2024 Nov 28;25(23):12803. doi: 10.3390/ijms252312803 (PMC11641184; doi:10.3390/ijms252312803)
Supplement: Supplementary file 1 [file ijms-25-12803-s001.zip › ijms-3343998-supplementary.pdf]

Supplementary material

Coumarin Derivative Hybrids: Novel Dual Inhibitors Targeting Acetylcholinesterase and Monoamine Oxidases for Alzheimer's Therapy

Teresa Żołek[1,*], Rosa Purgatorio[2], Łukasz Kłopotowski[1], Marco Catto[2] and Kinga Ostrowska[1]

[1]Department of Organic and Physical Chemistry, Faculty of Pharmacy, Medical University of Warsaw, Banacha 1, 02-097 Warsaw, Poland
[2]Department of Pharmacy-Pharmaceutical Sciences, University of Bari Aldo Moro, Via E. Orabona 4, 70125 Bari, Italy

(a)

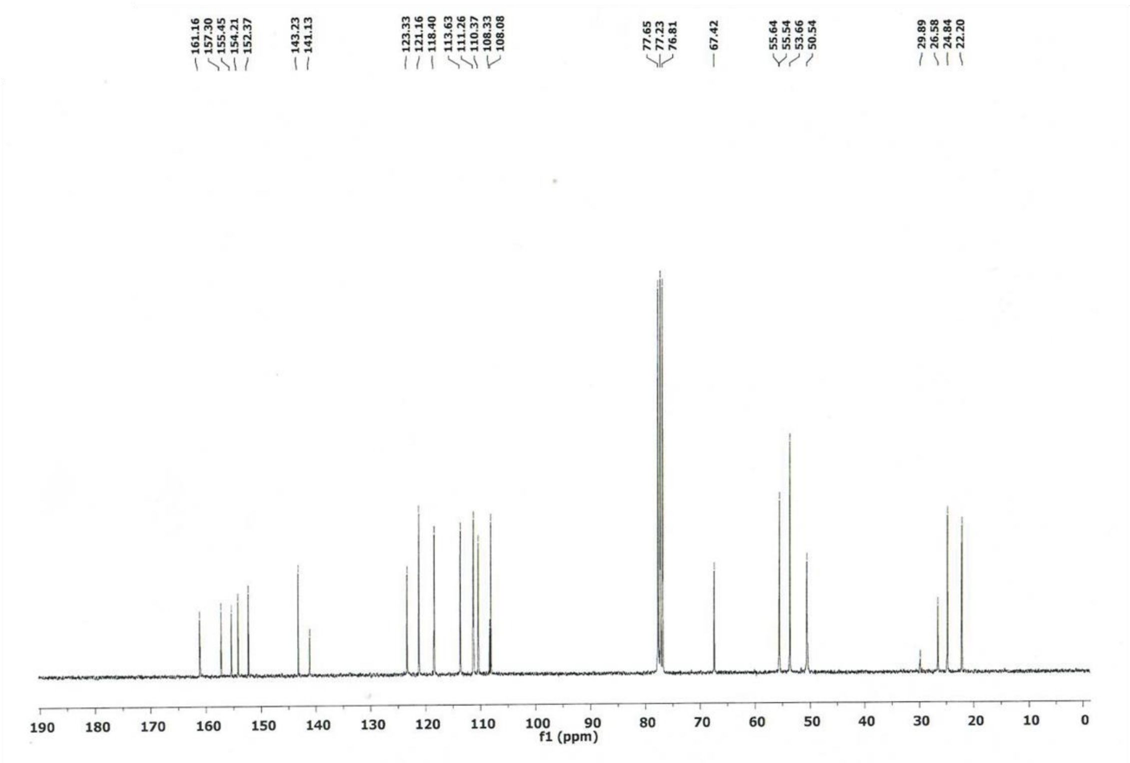

(b)

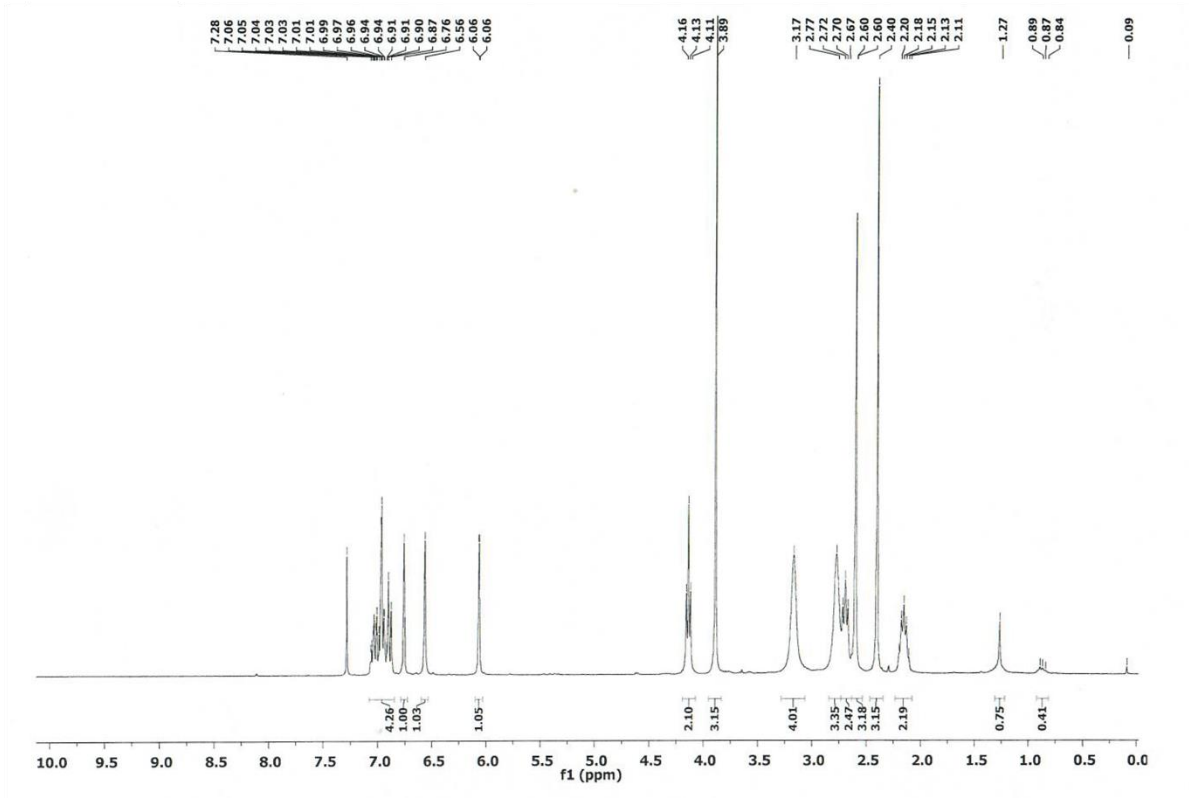

Figure S1. The spectra of 4,7-dimethyl-5-{3-[4-(2-methoxyphenyl)piperazin-1-yl]propoxy}coumarin (**1**). (**a**) ^{13}C CP/MAS NMR and (**b**) ^{1}H NMR.

(a)

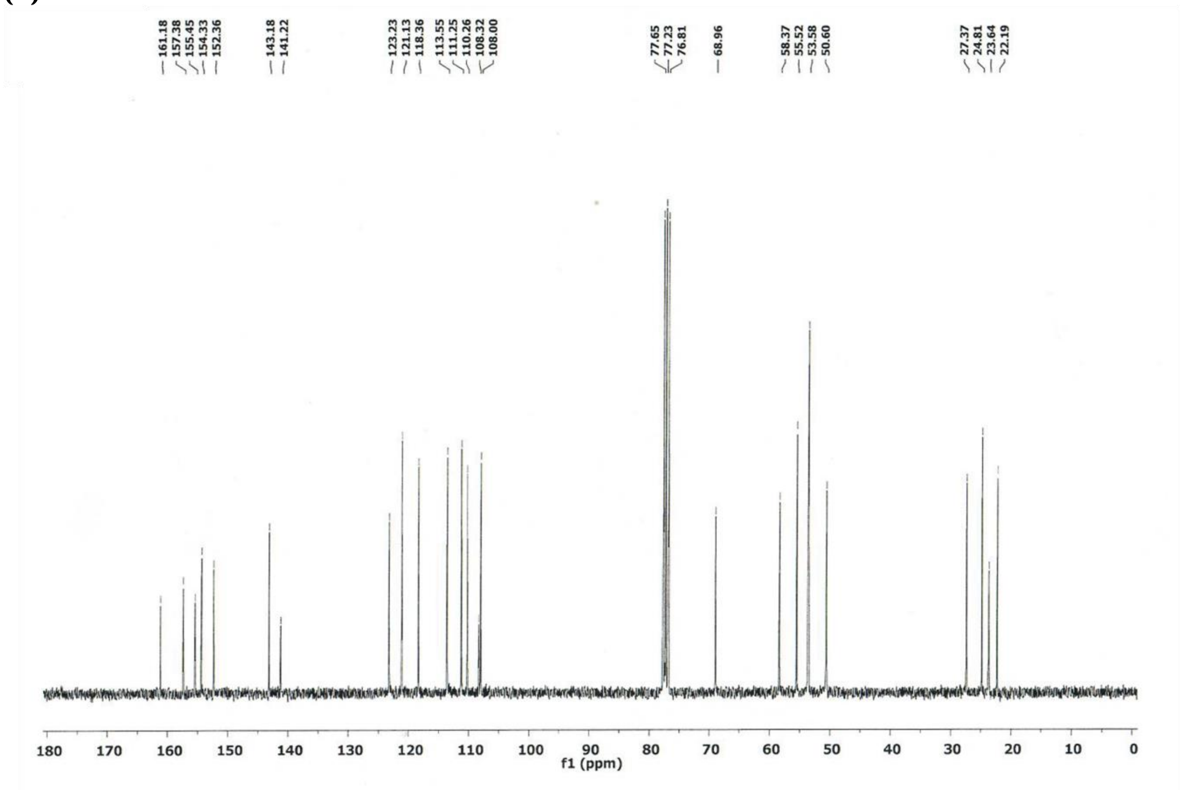

(b)

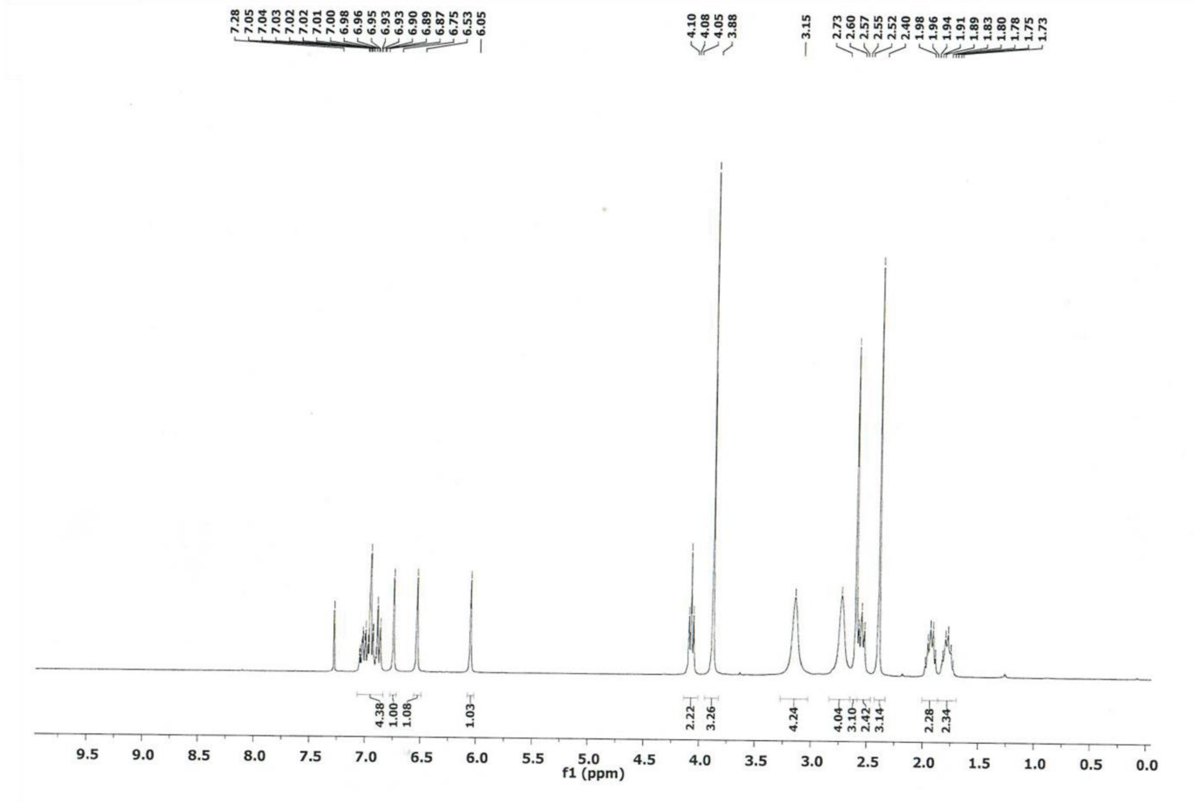

Figure S2. The spectra of 4,7-dimethyl-5-{4-[4-(2-methoxyphenyl)piperazin-1-yl]buthoxy}coumarin (**2**). (**a**) ^{13}C CP/MAS NMR and (**b**) ^{1}H NMR.
